# Development and Application of Extraction Methods for LC-MS Quantification of Microcystins in Liver Tissue

**DOI:** 10.3390/toxins12040263

**Published:** 2020-04-19

**Authors:** David Baliu-Rodriguez, Daria Kucheriavaia, Dilrukshika S. W. Palagama, Apurva Lad, Grace M. O’Neill, Johnna A. Birbeck, David J. Kennedy, Steven T. Haller, Judy A. Westrick, Dragan Isailovic

**Affiliations:** 1 Department of Chemistry and Biochemistry, University of Toledo, Toledo, OH 43606, USA; David.BaliuRodriguez@rockets.utoledo.edu (D.B.-R.); Daria.Kucheriavaia@rockets.utoledo.edu (D.K.); dilruksp@med.umich.edu (D.S.W.P.); 2 Department of Medicine, University of Toledo Medical Center, Toledo, OH 43614, USA; Apurva.Lad@rockets.utoledo.edu (A.L.); David.Kennedy@utoledo.edu (D.J.K.); Steven.Haller@utoledo.edu (S.T.H.); 3 Department of Chemistry, Wayne State University, Detroit, MI 48202, USA; grace.o.neill@wayne.edu (G.M.O.); jbirbeck@chem.wayne.edu (J.A.B.); judy.westrick@wayne.edu (J.A.W.)

**Keywords:** microcystins, liver, quantification, solid-phase extraction, liquid chromatography-mass spectrometry

## Abstract

A method was developed to extract and quantify microcystins (MCs) from mouse liver with limits of quantification (LOQs) lower than previously reported. MCs were extracted from 40-mg liver samples using 85:15 (v:v) CH_3_CN:H_2_O containing 200 mM ZnSO_4_ and 1% formic acid. Solid-phase extraction with a C18 cartridge was used for sample cleanup. MCs were detected and quantified using HPLC-orbitrap-MS with simultaneous MS/MS detection of the 135.08 *m/z* fragment from the conserved Adda amino acid for structural confirmation. The method was used to extract six MCs (MC-LR, MC-RR, MC-YR, MC-LA, MC-LF, and MC-LW) from spiked liver tissue and the MC-LR cysteine adduct (MC-LR-Cys) created by the glutathione detoxification pathway. Matrix-matched internal standard calibration curves were constructed for each MC (R^2^ ≥ 0.993), with LOQs between 0.25 ng per g of liver tissue (ng/g) and 0.75 ng/g for MC-LR, MC-RR, MC-YR, MC-LA, and MC-LR-Cys, and 2.5 ng/g for MC-LF and MC-LW. The protocol was applied to extract and quantify MC-LR and MC-LR-Cys from the liver of mice that had been gavaged with 50 µg or 100 µg of MC-LR per kg bodyweight and were euthanized 2 h, 4 h, or 48 h after final gavage. C57Bl/6J (wild type, control) and Lepr^db^/J (experiment) mice were used as a model to study non-alcoholic fatty liver disease. The Lepr^db^/J mice were relatively inefficient in metabolizing MC-LR into MC-LR-Cys, which is an important defense mechanism against MC-LR exposure. Trends were also observed as a function of MC-LR gavage amount and time between final MC-LR gavage and euthanasia/organ harvest.

## 1. Introduction

Microcystins (MCs) are toxic cyclic heptapeptides produced by cyanobacteria, which can occur in significant quantities in water during harmful algal blooms (HABs) [1,2,3]. These toxins have been reported in all inhabited continents [2,4,5,6,7,8] and are shown to induce hepatotoxicity and hepatocarcinogenesis [7,9,10,11]. The primary mechanism of toxicity is derived from the distinctive amino acid Adda (3-amino-9-methoxy-2,6,8-trimethyl-10-phenyldeca-4,6-dienoic acid) [12,13]. Over 250 MC congeners have been identified [14] and have the common name designation that specifies the amino acids in positions two and four (Figure S1) [13]. MCs may also differ by side-chain modifications and N-methylation. 

MCs are potent liver toxins that inhibit the function of protein phosphatases 1 and 2A (PP1 and PP2A) by binding to the enzymes’ active sites [15,16,17,18,19]. A covalent bond is formed between the active site of the enzyme and the Mdha residue of the MC via a Michael addition, and the enzyme is unable to dephosphorylate proteins [15]. The enzyme glutathione S-transferase bonds the MC to the cysteine (Cys) in glutathione (GSH) at the same Mdha site, forming the adduct MC-GSH (Figure S2A) [20,21]. MC-GSH is short-lived, however, and rapidly converts into MC-Cys (Figure S2B) [22]. MC-GSH and MC-Cys adducts are more readily excreted in urine due to their higher solubility [20,23].

Large MC doses lead to acute liver failure [7,9], but prolonged exposure to low levels of MCs may be more prevalent and pernicious. The effects of such exposure in humans are not well understood and are generally extrapolated from animal models [24,25,26,27,28]. Animal studies have found biomarkers of MC exposure in mouse serum [29] and rat urine [30] after intraperitoneal injection. Lepr^db^/J mice have been used to model non-alcoholic fatty liver disease (NAFLD) in humans, and our recent report in the Lepr^db^/J model of pre-existing NAFLD has shown that MC-LR exposure in those mice was found to exacerbate hepatic injury, even when MC-LR doses approximate the no observed adverse effect level (NOAEL) [24]. NAFLD is estimated to affect 26% of Americans and is predicted to increase in the US and abroad [31]. Further research is necessary to identify and diagnose MC exposure in human populations, and to understand the adverse effects of MC exposure in healthy and diseased livers.

MCs in tissue can be quantified using enzyme-linked immunosorbent assays (ELISA) [32], protein phosphatase inhibition assays (PPIA) [20,33], Lemieux oxidation (also known as MMPB) method [34,35], and LC-MS [22,36,37,38]. ELISA, PPIA, and Lemieux oxidation methods cannot distinguish between different MC congeners and adducts. Elucidating MC congener distribution is important because different congeners vary significantly in toxicity, and detection of MC adducts is necessary to study the metabolism and detoxification of MCs in tissue. Furthermore, a combination of poor recoveries, extensive sample preparation, and inadequate limits of detection (LODs) hinder the convenience and efficacy of those methods. LC-MS is robust and precise, but published methods for extraction of MCs from tissue have been validated for few congeners [36,37] or require complex sample preparation [38]. The lowest limit of quantification (LOQ) achieved previously, 17 ng of MC-LR per g of dried liver tissue [22], may not be adequate to study the effects of chronic exposure to MCs in low doses. Therefore, it is necessary to develop a simplified extraction method coupled with an accurate and precise LC-MS method that can quantify a variety of MC congeners and adducts in low concentrations in a liver tissue matrix.

A method was optimized to simultaneously extract six common MCs (MC-LR, MC-RR, MC-YR, MC-LA, MC-LF, and MC-LW) from mouse liver tissue using a novel extraction solvent composition. The method was also used to extract the MC-LR-Cys adduct. Sample cleanup was performed with solid-phase extraction (SPE), and MCs were separated and quantified using LC-orbitrap-MS. The method was used to quantify MC-LR and MC-LR-Cys in the liver of Lepr^db^/J and wild-type mice that were gavaged with MC-LR. Trends in concentration of MC-LR and MC-LR-Cys are shown to be dependent on mouse phenotype, amount of MC-LR administered, and time between final MC-LR administration and organ harvest.

## 2. Results and Discussion

### 2.1. Extraction Optimization

The extraction procedure was optimized by spiking MCs into liver samples and using liquid extraction to isolate MCs from tissue. Sample cleanup and pre-concentration were performed by SPE with a C18 cartridge, and LC-electrospray ionization (ESI)-selected-ion monitoring (SIM)-MS was used to separate MCs and measure peak areas of their monoisotopic ions. Monoisotopic masses of MC ions were determined with excellent mass accuracies (≤1.65 ppm; Table S1) using orbitrap. Retention times were approximately 5.39 min for MC-RR, 5.54 min for MC-LR-Cys, 5.91 min for MC-YR, 5.97 min for MC-LR, 6.79 min for C_2_D_5_ MC-LR, 7.71 min for MC-LA, 8.86 min for MC-LW, and 9.18 min for MC-LF (Figure S3). During optimization, all solvents and procedures were kept the same with the exception of the extraction solvent. Therefore, the difference in results from optimization experiments could be attributed to the extraction solvent used.

**Figure 1:**
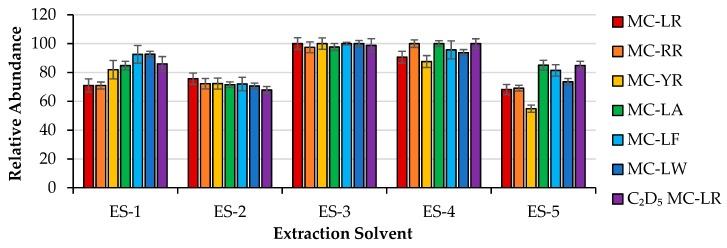
Relative abundances of seven microcystins (MCs) spiked into five mouse liver samples and extracted using different solvents: 85:15 (v:v) CH_3_OH:H_2_O (ES-1); 85:15 (v:v) CH_3_OH:H_2_O containing 100 mM ZnSO_4_ and 1% FA (ES-2); 85:15 (v:v) CH_3_CN:H_2_O containing100 mM ZnSO_4_ and 1% FA (ES-3); H_2_O containing 100 mM ZnSO_4_ and 1% FA followed by CH_3_CN containing 1% FA (ES-4); and H_2_O containing 0.01M EDTA, 3M NaCl, and 5% acetic acid followed by CH_3_OH (ES-5). Relative abundances were compared. Error bars are ± standard deviation of triplicate LC-MS measurements.

MC extraction was optimized for MC-LR, MC-RR, MC-YR, MC-LA, MC-LF, MC-LW, and C_2_D_5_ MC-LR. MC-LR-Cys was not used in extraction optimization experiments due to limited availability of the compound. Figure 1 shows the relative abundances of the seven MCs obtained from the first set of optimization experiments. Absolute recoveries were not calculated. Instead, extractions were compared relative to each solvent used per set of experiments. The peak areas of each monoisotopic MC ion were determined using triplicate LC-MS measurements. Each congener was then considered separately; the largest peak area of each congener was considered a standard and assigned a relative abundance of 100%. The smaller peak areas of the same MC congener were divided by the standard and plotted alongside the other congeners extracted using the same extraction solvent. This allowed for a relative measurement of the extraction efficiency without the need for determination of absolute recovery. 

Five extraction solvents were used for initial optimization experiments: 85:15 (v:v) CH_3_OH:H_2_O (ES-1); 85:15 (v:v) CH_3_OH:H_2_O containing 100 mM ZnSO_4_ and 1% FA (ES-2); 85:15 (v:v) CH_3_CN:H_2_O containing 100 mM ZnSO_4_ and 1% FA (ES-3); H_2_O containing 100 mM ZnSO_4_ and 1% FA, followed by CH_3_CN containing 1% FA (ES-4); and H_2_O containing 0.01M EDTA, 3M NaCl, and 5% acetic acid, followed by CH_3_OH (ES-5). MCs were extracted from each spiked liver sample as described in Section 4.4. For ES-4 and ES-5, different solvents were used for the final two extractions. Solvents ES-1 and ES-5 were chosen based on previous studies concerning extraction of MCs from animal tissues [22,36]. Three solvents with ZnSO_4_ (ES-2, ES-3, and ES-4) were chosen for extraction of MCs from liver because zinc binds to an estimated 3000 human proteins [39], including PP1 [40] and PP2A [41], and potentially displaces noncovalently bound MCs or inhibits MCs from binding. ZnSO_4_ has also been used to improve extraction of MCs from plasma and serum under acidic conditions [42]. 

Figure 1 shows that solvent ES-3 (85:15 (v:v) CH_3_CN:H_2_O containing 100 mM ZnSO_4_ and 1% FA) was the most effective, with relative abundance ≥ 97% for all seven congeners. Results from ES-3 and ES-4 indicate that the combination of ZnSO_4_ and CH_3_CN under acidic conditions are most effective for MC extraction, and that the solvents are more effective when used in tandem, as opposed to sequentially. However, CH_3_OH:H_2_O with and without acidified ZnSO_4_ (ES-1 and ES-2) were not as effective as CH_3_CN:H_2_O with acidified ZnSO_4_. Previous work shows that CH_3_CN is more effective than CH_3_OH for elution of MCs from C18 SPE cartridges [43], and similar trends were observed here. Extraction of MCs using water with high salt concentration instead of an organic solvent was not successful and had the lowest recoveries for the more polar MCs (MC-LR, MC-RR, and MC-YR). 

ES-3 was used as a basis for further MC extraction, and different factors of its composition were tested to optimize its capabilities. The ideal CH_3_CN:H_2_O ratio was tested in the next set of experiments. The solvents prepared were 55:45, 65:35, 75:25, 85:15, and 100:0 (v:v) CH_3_CN:H_2_O. Each solvent was acidified with 1% FA and labelled ES-6 through ES-10, respectively. Extraction efficiencies were compared as described previously, and results are shown in Figure S4. ES-9 had the best recoveries, with relative abundances ≥ 97% for all MCs besides C_2_D_5_ MC-LR. Generally, the extraction of more polar MCs did not vary much with a change in CH_3_CN:H_2_O ratio as long as the solvent contained some amount of H_2_O. However, extraction of the less polar MCs (MC-LA, MC-LW, and MC-LF) dropped significantly when the CH_3_CN content was less than 85%. This trend indicates that their extraction improves as polarity of the extraction solvent decreases. The extraction solvent ES-9 (85:15 (v:v) CH_3_CN:H_2_O containing 1% FA) was used for further experiments. 

The changes caused by varying the acid and ZnSO_4_ content of ES-9 were tested in the next set of experiments. Four solvents were used—85:15 (v:v) CH_3_CN:H_2_O containing 0.1% FA (ES-11), 85:15 (v:v) CH_3_CN:H_2_O containing 1% FA (ES-9), 85:15 (v:v) CH_3_CN:H_2_O containing 100 mM ZnSO_4_ and 0.1% FA (ES-12), and 85:15 (v:v) CH_3_CN:H_2_O containing 100 mM ZnSO_4_ and 1% FA (ES-3). Results are shown in Figure S5. Trends indicate that the addition of ZnSO_4_ improves extraction of more polar MCs. ES-3 was most successful in extracting those MCs, with relative abundances ≥ 98% for MC-LR, MC-RR, and MC-YR. MC-LA and C_2_D_5_ MC-LR were also extracted most efficiently by ES-3, though MC-LF and MC-LW had relative abundances of 91% and 85%, respectively. ES-11 had relative abundances ≥ 98% for MC-LA, MC-LF, and MC-LW, with relative abundances ≥ 87% for the remaining MCs. Since MC-LR and MC-RR are reported to be the most common in nature [16], those congeners are the most important to extract and extraction optimization was continued using ES-3.

The final optimization step was to determine how much ZnSO_4_ should be used. Five solvents were prepared with varying amounts of ZnSO_4_: 85:15 (v:v) CH_3_CN:H_2_O containing 25 mM ZnSO_4_ and 1% FA (ES-13), 85:15 (v:v) CH_3_CN:H_2_O containing 50 mM ZnSO_4_ and 1% FA (ES-14), 85:15 (v:v) CH_3_CN: H_2_O containing 100 mM ZnSO_4_ and 1% FA (ES-3), 85:15 (v:v) CH_3_CN:H_2_O containing 150 mM ZnSO_4_ and 1% FA (ES-15), and 85:15 (v:v) CH_3_CN:H_2_O containing 200 mM ZnSO_4_ and 1% FA (ES-16). Five 20-mg liver samples were spiked with 7 MCs, and the MCs were extracted and detected using the method described in Section 4.4. Results are shown in Figure S6. Clear trends can be seen as ZnSO_4_ content increases. MC-LR, MC-YR, MC-LA, and C_2_D_5_ MC-LR are extracted more efficiently with more zinc, though MC-LF and MC-LW are best extracted with ≤ 100 mM ZnSO_4_ concentration. MC-RR, the most polar MC, is extracted well by all five solvents. ES-16 was the optimal solvent for extraction of MCs from tissue, and was used for recovery and quantification experiments.

### 2.2. Recovery Experiments

**Figure 2:**
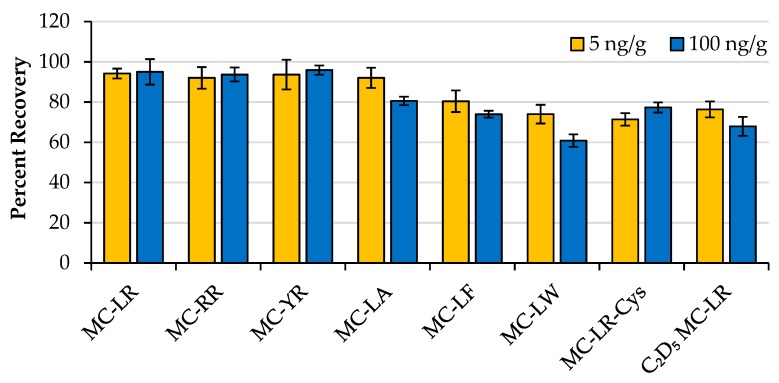
Percent recoveries of eight MCs spiked into 20-mg mouse liver samples and extracted using the optimized procedure. Recoveries were determined by performing the extraction procedure on control mouse liver samples and spiking with MCs prior to LC-MS analysis. Three replicate samples were analyzed at low and high concentration levels. Error bars are ± standard deviation of the average recovery of the replicate samples.

Three 20-mg wild-type mouse liver samples were spiked with eight MCs at a low concentration (5 ng/g), three were spiked at a high concentration (100 ng/g), and the MCs were extracted using the optimized procedure. Two controls were prepared by performing the extraction procedure on wild-type liver samples that did not contain MCs. The controls were spiked with 5 ng/g or 100 ng/g of eight MCs prior to LC-MS analysis, and the eight samples were analyzed sequentially in triplicate. Recovery was determined by dividing the average peak area of the three replicate samples by the average peak area of the corresponding control sample for each monoisotopic MC ion. This signal comparison measured the loss of each MC congener during the extraction procedure and allowed for an absolute measure of extraction efficiency. Results are shown in Figure 2 and Table S2. MC-LR, MC-RR, and MC-YR had recoveries ≥ 92.0% at both concentration levels. Recoveries for MC-LA, MC-LF, MC-LW, MC-LR-Cys, and C_2_D_5_ MC-LR were ≥ 80.6%, 74.0%, 60.8%, 71.4%, and 67.9%, respectively. Recovery experiments were preferential for MC-LR because it is the most frequently reported congener, and MC-LR was the cyanotoxin used in the NAFLD mouse model study.

### 2.3. Calibration Curves and LOQs

Calibration curves were prepared separately for MCs extracted from wild-type and Lepr^db^/J mouse liver samples. Forty-milligram liver samples were spiked with seven MCs at 11 concentration levels ranging from 0.25 ng/g to 250 ng/g, though not all calibration curves had 11 points due to variable LOQs. The range was chosen to accommodate the broad MC concentrations that may be found in the actual samples. The liver samples were also spiked with the internal standard C_2_D_5_ MC-LR prior to tissue lysis. After extraction and LC-MS analysis, internal standard calibration curves were constructed by plotting the concentration of MC divided by the concentration of the standard on the x-axis, and the peak area of the monoisotopic MC ion divided by the peak area of the standard on the y-axis. Internal standard calibration curve equations, R^2^ values, and LOQs are shown in Table 1, and calibration curves are shown in Figure S7. 

The R^2^ values for the calibration curves of MC-LR, MC-RR, MC-YR, MC-LA, MC-LW, and MC-LR-Cys were ≥ 0.995, indicating excellent linearity across the calibration curve range. The R^2^ for MC-LF was ≥ 0.993. LOQs ranged from 0.25 ng/g to 2.5 ng/g depending on congener. LOQs were determined based on the US FDA Bioanalytical Method Validation guidelines [44], in which the lowest point on the calibration curve with error and RSD ≤ 20% is accepted as the LOQ. These data are shown in Table S3. LOQs for MC-LR, MC-RR, and MC-YR were 0.25 ng/g or 0.50 ng/g, and MC-LA, MC-LF, MC-LW, and MC-LR-Cys had LOQs of 0.75 ng/g or 2.50 ng/g (Table 1). Less polar MCs are shown to have higher LOQs when extracted from water [43] due to poorer ionizability than MCs that contain arginine, and this difference was observed in MCs extracted from liver samples. Another factor to consider is the complexity of the tissue matrix even after sample purification. Peptides and other molecules in the matrix may have ion suppressing effects or may contribute significantly to background noise that increases the LOQ of coeluting MCs [45]. Furthermore, the singly charged ions of MC-RR and MC-LR-Cys were not considered for quantification. This may explain why MC-RR and MC-LR-Cys have higher LOQs than MC-LR, despite the presence of an additional primary amine or guanidine. Further sample purification may minimize matrix effects, but sample preparation should strike a balance between purity, cost-effectiveness, and complexity of sample preparation.

### 2.4. Quantification of MC-LR and MC-LR-Cys in Livers of Gavaged Mice

Unbound MC-LR and MC-LR-Cys were quantified in the liver of mice that were gavaged with MC-LR. Liver samples weighing 40 mg were used instead of 20 mg to increase the amount of MCs in the sample. In order to accommodate the larger sample size, the amount of extraction solvent was doubled from 3 mL to 6 mL, and extractions were performed as detailed in Section 4.5. 

MCs were quantified in the liver of wild-type mice labelled 1–15 using the calibration curve prepared with wild-type mouse liver samples. The wild-type mice were gavaged with 100 µg of MC-LR per kg bodyweight. Mice 1–5 were sacrificed two hours after final gavage, mice 6–10 were sacrificed four hours after final gavage, and mice 11–15 were sacrificed 48 h after final gavage. Results from the experiment are shown in Table 2 and Figure S8. MC-LR was detected but could not be quantified due to low signal in mice 2 and 9. 

MC-LR and MC-LR-Cys concentrations had different trends as a function of time after final gavage. The average MC-LR concentrations decreased from 0.81 ng/g to 0.68 ng/g to 0.33 ng/g for the 2, 4, and 48 h sets, respectively. The average MC-LR-Cys concentrations were 38.43, 90.18, and 52.04 ng/g for the 2, 4, and 48 h sets, respectively. MC-LR-Cys concentrations were two orders of magnitude larger than MC-LR concentrations in the wild-type mice, indicating the efficiency with which MC-LR in the liver is converted to MC-LR-GSH, which rapidly metabolizes to MC-LR-Cys. The trend for MC-LR-Cys concentration as a function of time shows an increase in MC-LR-Cys concentration from 2 h to 4 h, followed by a decrease from 4 h to 48 h. The drop in MC-LR-Cys concentration is likely due to expulsion of the Cys adduct from the liver, as MC-LR-Cys is more soluble and easier to excrete via urine [20,23]. These trends follow expectations, as time allows for the toxin to be metabolized and removed from the liver.

Lepr^db^/J mouse liver samples were used to construct the calibration curve used for quantification of MC-LR and MC-LR-Cys in the livers of 16 Lepr^db^/J mice. Mice 1–9 were gavaged with 50 µg MC-LR per kg bodyweight. Mice 1–4 and mice 5–9 were euthanized 2 and 4 h after final gavage, respectively. Mice 10–16 were gavaged with 100 µg MC-LR per kg bodyweight. Mice 10–13 and mice 14–16 were euthanized 2 and 4 h after final gavage, respectively. Results are shown in Table 3 and Figure S9. The MC-LR-Cys concentration in mouse 12 was 45.75 ng/g, more than 5 times greater than the next highest value from the same set of mice. The data point was found to be an outlier using Grubbs’ test with 95% confidence, and removed. It is possible that the 40-mg liver sample obtained from mouse 12 was not representative of the whole liver. A pocket of adipocytes or other cells high in MC-LR-Cys may have been analyzed, thus misrepresenting the concentration of the Cys adduct in the whole liver.

As in the wild-type mouse liver samples, the average concentrations of MC-LR decreased from 2–4 h, and the average concentrations of MC-LR-Cys increased in the same time frame. However, those differences are not as obvious as in wild-type samples and further studies involving larger numbers of samples are needed to elucidate those trends. No liver samples were obtained from Lepr^db^/J mice sacrificed 48 h after final gavage, so it may be expected though not confirmed that the MC-LR-Cys concentration would have decreased at the 48-h time point.

There were significant differences in the metabolism of MC-LR between wild-type and Lepr^db^/J mice. MC-LR concentration was much lower in wild-type mice at the same time points, and MC-LR-Cys concentration was higher, indicating that wild-type mice were converting MC-LR into MC-LR-Cys more efficiently. This finding correlates with expectations, as Lepr^db^/J mice are used to model NAFLD.

## 3. Conclusion

The extraction and quantification of MC congeners and adducts from tissue is imperative in understanding the toxicity of MCs. Herein, a method was developed and optimized that allows for a broad range of common MC congeners and a MC-LR adduct (MC-LR, MC-RR, MC-YR, MC-LA, MC-LF, MC-LW, and MC-LR-Cys) to be simultaneously extracted from liver tissue with recoveries ranging from 60.1% to 95.9%. MCs were detected and quantified using LC-SIM-high-resolution-MS with LOQs between 0.25 ng/g and 2.5 ng/g, which is two orders of magnitude lower than previously reported [22]. 

The method was applied in a proof-of-concept study to examine the effect of MC exposure on liver function in mice with NAFLD. MC-LR and MC-LR-Cys were quantified in the liver of wild-type and Lepr^db^/J mice that were gavaged with MC-LR, and trends were observed based on mouse phenotype, MC-LR dose, and time between final MC-LR administration and euthanasia. It was discovered that Lepr^db^/J mice are relatively inefficient in metabolizing MC-LR into MC-LR-Cys, which is an important defense mechanism against the toxin. This work provides a basis for further experiments that investigate MCs and MC adducts found in liver tissue of affected animals, and it is expected that the method works efficiently for the extraction of MCs from other tissues as well. 

## 4. Materials and Methods

### 4.1. Reagents

MC-LR, MC-RR, and MC-LA were purchased from Cayman Chemical (Ann Arbor, MI, USA). MC-YR, MC-LF, and MC-LW were purchased from Enzo Life Sciences (Farmingdale, NY, USA). C_2_D_5_ MC-LR was purchased from Cambridge Isotope Laboratories (Tewksbury, MA, USA). MC-LR-Cys was synthesized by the Westrick group (Wayne State University, Detroit, MI, USA) [46]. ACS-grade zinc sulfate (ZnSO_4_) and HPLC-grade water, methanol (CH_3_OH), and acetonitrile (CH_3_CN) were purchased from Fisher Scientific (Pittsburgh, PA, USA). Reagent-grade formic acid (FA) was purchased from Sigma (St. Louis, MO, USA).

### 4.2. Materials and Instruments

Sep-Pak Light C18 cartridges with 130 mg sorbent beds were purchased from Waters (Milford, MA, USA). Three and 10 mL syringes were purchased from Becton, Dickinson and Company (Franklin Lakes, NJ, USA). Two milliliter clear glass vials and 100 µL glass inserts were purchased from Sigma (St. Louis, MO, USA). Two milliliter centrifuge tubes were purchased from Fisher Scientific (Pittsburgh, PA, USA). The centrifuge and vacuum concentrator were from Eppendorf (Hamburg, Germany). Tissue samples were lysed using a Qiagen TissueLyzer II (Hilden, Germany). Stainless steel beads (5 mm) were purchased from Qiagen.

Separation of MCs was achieved using a Shimadzu (Addison, IL, USA) HPLC instrument consisting of a CBM-20A system controller, two LC-20AD pumps, a DGU-20A3 degasser, and a SIL-20A HT autosampler. The chromatographic column was a Waters XBridge C8 (3.0 × 100 mm, 3.5 µm particle size) preceded by an XBridge C8 guard column (3.0 × 20 mm, 3.5 µm particle size). MCs were detected and quantified using a Thermo (San Jose, CA, USA) Orbitrap Fusion Tribrid mass spectrometer equipped with a heated ESI source. 

### 4.3. Mouse Liver Collection 

All animal experiments were conducted in accordance with the National Institute of Health Guide for the Care and Use of Laboratory Animals using protocols approved by the Institutional Animal Use and Care Committee at the University of Toledo Health Science Campus (IACUC protocol number 108663, approval date 9 February 2016). Eight-week-old male B6.BKS(D)-Lepr^db^/J (JAX Stock No. 000697, B6 db) mice (referred to as Lepr^db^/J mice) on the C57Bl/6J background and C57Bl/6J (JAX Stock No. 000664, Black 6) healthy background strain control mice (referred to as wild-type mice) were purchased from The Jackson Laboratory (Bar Harbor, ME, USA) and maintained in the Department of Laboratory Animal Research at University of Toledo. All animals were specific-pathogen free and were housed in plastic cages (five mice per cage) and fed ad libitum on balanced rodent diet (Teklad global 16% protein diet, Envigo, Indianapolis, IN, USA) and water. The mice were kept in a well-ventilated room maintained at 23 ± 1 °C on a 12-h light and dark cycle. The animals were acclimatized for a week prior to the study. 

At 10-weeks of age, the Lepr^db^/J mice, each weighing around 40–45 g, were divided into three groups. Mice treated with vehicle received 300 µL of Milli-Q water, while the other two groups received 300 µL of a solution containing 50 µg or 100 µg MC-LR per kg bodyweight. The wild-type mice, each weighing around 20–25 g, were divided into two groups and treated with vehicle or 100 µg MC-LR per kg bodyweight. MC-LR stock solution of 0.5 mg/mL was made in Milli-Q water and was further diluted to the required dose. All mice were gavaged with water or MC-LR solution every 48 h for a total of four weeks (15 dose administrations). These doses approximate the currently accepted NOAEL of 40 µg MC-LR per kg bodyweight established after 13-week MC-LR administration [26]. The mice were euthanized 2, 4, or 48 h after final gavage, and the harvested livers were stored at −80 °C. Wet mass was used for liver sample measurements. The mass of each liver sample used for optimization and recovery experiments was 20 ± 1 mg, while the mass of each liver sample used for calibration curves and quantitative analysis was 40 ± 1 mg. 

### 4.4. Sample Preparation for Optimization and Recovery Experiments

Liver samples weighing 20 ± 1 mg from wild-type mice gavaged with vehicle were placed in a 2 mL centrifuge tube and spiked with a solution of MCs in water. A stainless steel bead and 1 mL of the extraction solvent were added to the centrifuge tube, and the sample was homogenized by a tissue lyser set at 25 Hz for 10 min. The sample was sonicated in a bath for 3 min at 0 °C and centrifuged for 5 min at 10,000 rpm. The supernatant was collected in a 2 mL glass vial and the extraction procedure was repeated on the pellet two more times. The supernatants were combined in the glass vial and the solvent was evaporated using a vacuum concentrator. 

The remaining content of the glass vial was reconstituted in 3 mL of water and transferred to a 50 mL centrifuge tube containing 2 mL of water. SPE was performed on the sample using a Sep-Pak C18 cartridge as follows: the cartridge was conditioned and equilibrated using 2 mL of 90:10 (v:v) CH_3_OH:H_2_O containing 0.1% FA and 2 mL of H_2_O containing 0.1% FA, respectively. The 5 mL sample was drawn from the 50 mL centrifuge tube using a 10 mL syringe and transferred onto the SPE cartridge. The cartridge was washed with 2 mL of H_2_O containing 0.1% FA, and the sample was eluted into a 2 mL glass vial using 1.75 mL of 90:10 (v:v) CH_3_CN:H_2_O containing 0.1% FA. The solvent was evaporated in a vacuum concentrator and reconstituted in 200 µL of 90:10 (v:v) CH_3_OH:H_2_O containing 0.1% FA for LC-MS analysis. 

Liver samples used for optimization experiments were spiked with 20 µL of an aqueous solution containing 250 µg/L of 7 MCs (MC-LR, MC-RR, MC-YR, MC-LA, MC-LF, MC-LW, and C_2_D_5_ MC-LR). Recovery experiments were performed at low and high concentrations by spiking liver samples with 20 µL of an aqueous solution containing 5 µg/L or 100 µg/L of each of 8 MCs (MC-LR, MC-RR, MC-YR, MC-LA, MC-LF, MC-LW, C_2_D_5_ MC-LR, and MC-LR-Cys) and extracting the MCs using the optimized procedure. Controls for recovery experiments were prepared by performing the optimized sample preparation procedure on liver samples that were spiked with 20 µL of a solution containing 5 µg/L or 100 µg/L of each MC immediately prior to LC-MS analysis. 

### 4.5. Sample Preparation for Calibration Curves and Livers of Mice Gavaged with MC-LR

To prepare a calibration curve, 40-mg liver samples from mice gavaged with vehicle were placed in a 2 mL centrifuge tube and spiked with a solution of MCs in water. A stainless steel bead and 1.5 mL of 85:15 (v:v) CH_3_CN:H_2_O containing 200 mM ZnSO_4_ and 1% FA was added to each centrifuge tube, and the samples were homogenized by a tissue lyser set at 25 Hz for 10 min. The samples were sonicated for 3 min at 0 °C and centrifuged for 5 min at 10,000 rpm. The supernatant of each sample was collected in a 2 mL glass vial and the extraction procedure was repeated on the pellet three more times. The supernatants were combined in the glass vial and the solvent was evaporated using a vacuum concentrator.

Once the supernatant was evaporated, the contents of each glass vial were reconstituted in 3 mL of water and transferred to a 50 mL centrifuge tube containing 2 mL of water. SPE was performed as described in Section 4.4. The elution solvent was evaporated using a vacuum concentrator, and the sample was reconstituted in 100 µL of 90:10 (v:v) CH_3_OH:H_2_O containing 0.1% FA for LC-MS analysis. 

Separate calibration curves were prepared for wild-type and Lepr^db^/J using control liver samples from each to quantify MCs. The liver samples were spiked from a range of 0.25 to 250 ng/g of each of 7 MCs: MC-LR, MC-RR, MC-YR, MC-LA, MC-LF, MC-LW, and MC-LR-Cys. The livers were spiked with 20 µL of a solution containing 20 µg/L of C_2_D_5_ MC-LR for use as an internal standard. Matrix-matched internal standard calibration curves were created for each MC from 0.25 ng/g to 250 ng/g. 

Liver samples weighing 40 mg from wild-type and Lepr^db^/J mice gavaged with MC-LR were spiked with 20 µL of a solution containing 20 µg/L of C_2_D_5_ MC-LR. MCs were extracted following the same protocol as for the calibration curves, and the calibration curves were used to quantify MC-LR and MC-LR-Cys in the liver of those mice.

### 4.6. Liquid Chromatography and Mass Spectrometry

Chromatography and mass spectrometry conditions were modified from a previously developed method [43]. A binary gradient was used for chromatography, with mobile phase A containing H_2_O with 0.05% FA and mobile phase B containing CH_3_CN with 0.05% FA. The C8 column was equilibrated at 20% B, and the gradient started at 20% B and was increased to 60% B from 0–2 min, increased to 70% B from 2–7 min, and increased to 90% B from 7–12 min. The gradient was then decreased to 20% B from 12–14 min and maintained at 20% B for 6 min. Chromatographic run time was 20 min, and 20 µL of sample was injected onto the column.

Mass spectrometry was performed with an Orbitrap Fusion Tribrid instrument equipped with quadrupole, orbitrap, and linear ion trap mass analyzers. Positively charged ions were formed with a heated ESI source. The quadrupole was used for SIM (5 *m/z* window) of the singly charged ions of MC-LR (995.56 *m/z*), MC-YR (1045.54 *m/z*), MC-LA (910.49 *m/z*), MC-LF (986.52 *m/z*), MC-LW (1025.53 *m/z*), and C_2_D_5_ MC-LR (1028.62 *m/z*), and for the doubly charged ions of MC-RR (519.79 *m/z*) and MC-LR-Cys (558.79 *m/z*). The orbitrap mass analyzer was used to detect MC ions with high mass accuracy (≤1.65 ppm; Table S1), and peak areas of the monoisotopic MC ions were obtained from the extracted ion chromatograms and used for quantification. Simultaneous MS/MS analysis was performed with the linear ion trap. MC ions were fragmented using higher-energy collision induced dissociation (HCD), and the characteristic Adda fragment ion with 135.08 *m/z* was used for structural confirmation. All samples were analyzed in triplicate, and results are reported as the average of three runs. Data were acquired and processed using Xcalibur software version 2.1 (Thermo Scientific, Waltham, MA, USA). 

## Figures and Tables

**Table 1:** Calibration curves, R^2^ values, limits of detection (LODs), and limits of quantification (LOQs) of seven MCs spiked into wild-type and Lepr^db^/J mouse liver samples and extracted using the optimized protocol.

MC Congener	LOD* (ng/g)	LOQ (ng/g)	Wild-type Calibration Curves		Lepr^db^/J Calibration Curves	
			Equation	R²	Equation	R²
MC-LR	0.08	0.25	y = 0.7133x + 0.0013	0.997	y = 1.2856x + 0.1187	0.995
MC-RR	0.15	0.50	y = 1.7273x − 0.0625	0.997	y = 2.0529x − 0.0126	0.995
MC-YR	0.15	0.50	y = 0.5487x + 0.0247	0.999	y = 0.5702x + 0.0057	0.998
MC-LA	0.23	0.75	y = 1.2411x − 0.0357	0.995	y = 0.9152x + 0.0077	0.997
MC-LF	0.75	2.50	y = 0.4632x − 0.1415	0.993	y = 0.4404x − 0.0193	0.994
MC-LW	0.75	2.50	y = 0.5882x − 0.0726	0.999	y = 0.4380x − 0.0005	0.999
MC-LR-Cys	0.23	0.75	y = 1.4500x − 0.1208	0.997	y = 2.4115x − 0.0030	0.999

*LOD estimated from three tenths of the LOQ.

**Table 2:** Concentrations of MC-LR and MC-LR-Cys in the liver of wild-type mice gavaged with 100 µg MC-LR per kg bodyweight.

Mouse	Time after Final Gavage	MC-LR Concentration (ng/g)			MC-LR-Cys Concentration (ng/g)		
		Individual	Average	Standard Deviation	Individual	Average	Standard Deviation
1	2 h	0.73	0.81	0.48	40.85	38.43	14.64
2		*			31.49		
3		0.44			23.18		
4		1.51			62.01		
5		0.56			34.63		
6	4 h	0.55	0.68	0.15	48.60	90.18	26.45
7		0.87			97.24		
8		0.75			81.32		
9		*			111.23		
10		0.56			112.49		
11	48 h	0.40	0.33	0.05	20.25	52.04	25.40
12		0.30			80.62		
13		0.35			33.94		
14		0.32			52.77		
15		0.26			72.63		

*MC-LR ions in the livers of mice 2 and 9 were detected below the LOQ and were not considered in calculations of the average concentration and standard deviation.

**Table 3:** Concentration of MC-LR and MC-LR-Cys in the liver of Lepr^db^/J mice gavaged with 50 µg or 100 µg MC-LR per kg bodyweight.

Mouse	MC-LR Dosage	Time after Final Gavage	MC-LR Concentration (ng/g)			MC-LR-Cys Concentration (ng/g)		
			Individual	Average	Standard Deviation	Individual	Average	Standard Deviation
1	50 µg/kg	2 h	14.21	12.64	5.37	1.17	2.77	1.38
2			8.37			4.38		
3			8.41			2.21		
4			19.57			3.31		
5		4 h	11.45	12.17	5.01	2.16	2.90	1.00
6			15.55			3.16		
7			14.51			4.15		
8			3.63			1.65		
9			7.18			3.41		
10	100 µg/kg	2 h	12.95	16.38	3.96	5.10	7.23	2.03
11			14.78			7.44		
12			22.05			*		
13			15.72			9.14		
14		4 h	18.54	13.50	4.42	13.83	11.24	2.37
15			10.29			9.16		
16			11.66			10.75		

*MC-LR-Cys concentration in the liver of mouse 12 was found to be an outlier using Grubbs’ test with 95% confidence.

## Supplementary Materials

The following are available online at https://www.mdpi.com/2072-6651/12/4/263/s1, Figure S1. General MC structure.; Figure S2. General structures of common MC adducts.; Figure S3. LC-SIM-MS chromatograms showing separation of eight MCs.; Figure S4. Relative abundances of MCs extracted using solvents ES-6, ES-7, ES-8, ES-9, and ES-10.; Figure S5. Relative abundances of MCs extracted using solvents ES-11, ES-9, ES-12, and ES-3.; Figure S6. Relative abundances of MCs extracted using solvents ES-13, ES-14, ES-3, ES-15, and ES-16.; Figure S7. Calibration curves used to quantify 7 MCs extracted from wild-type and Lepr^db^/J mouse liver samples.; Figure S8. Concentration of MC-LR and MC-LR-Cys in the livers of wild-type mice gavaged with MC-LR.; Figure S9. Concentration of MC-LR and MC-LR-Cys in the livers of Lepr^db^/J mice gavaged with MC-LR.; Table S1. Mass accuracies of detected monoisotopic MC ions.; Table S2. Percent recoveries of eight MCs spiked at two concentration levels into 20-mg mouse liver samples and extracted using the optimized procedure.; Table S3. Percent errors and relative standard deviations at the LOQs of seven MCs.
