# Supplementary material for: Development and Application of Extraction Methods for LC-MS Quantification of Microcystins in Liver Tissue
Source: Toxins (Basel). 2020 Apr 19;12(4):263. doi: 10.3390/toxins12040263 (PMC7232250; doi:10.3390/toxins12040263)
Supplement: Supplementary file 1 [file toxins-12-00263-s001.pdf]

Supplementary Materials: Development and Application of Extraction Methods for LC-MS Quantification of Microcystins in Liver Tissue

David Baliu-Rodriguez, Daria Kucheriavaia, Dilrukshika S. W. Palagama, Apurva Lad, Grace M. O'Neill, Johnna A. Birbeck, David J. Kennedy, Steven T. Haller, Judy A. Westrick and Dragan Isailovic

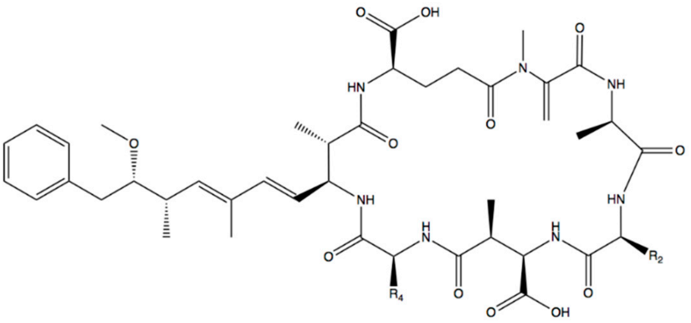

Congener	2	4
MC-LR	Leu	Arg
MC-RR	Arg	Arg
MC-YR	Tyr	Arg
MC-LA	Leu	Ala
MC-LW	Leu	Trp
MC-LF	Leu	Phe

Figure S1. General MC structure. Amino acids at positions 2 and 4 vary depending on congener. Common congeners are shown in the inset.

(**A**)

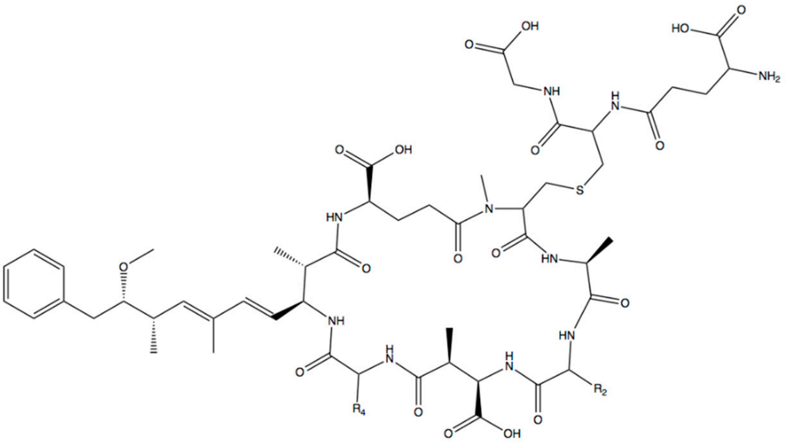

(**B**)

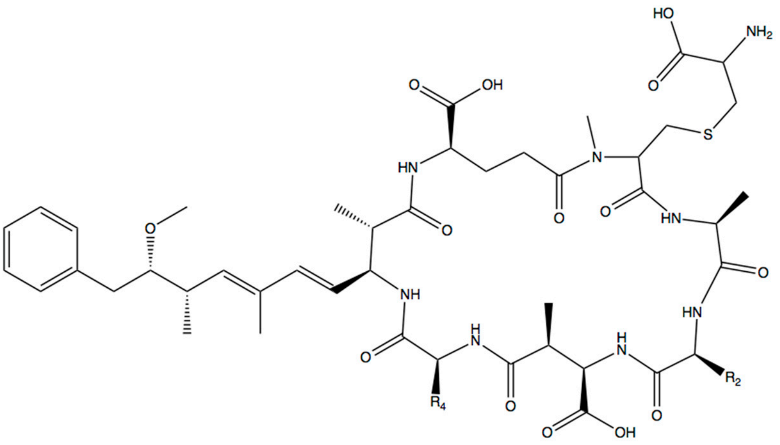

Figure S2. General structures of common MC adducts **A**) MC-GSH and **B**) MC-Cys. Primary amines are protonated at mobile phase pH.

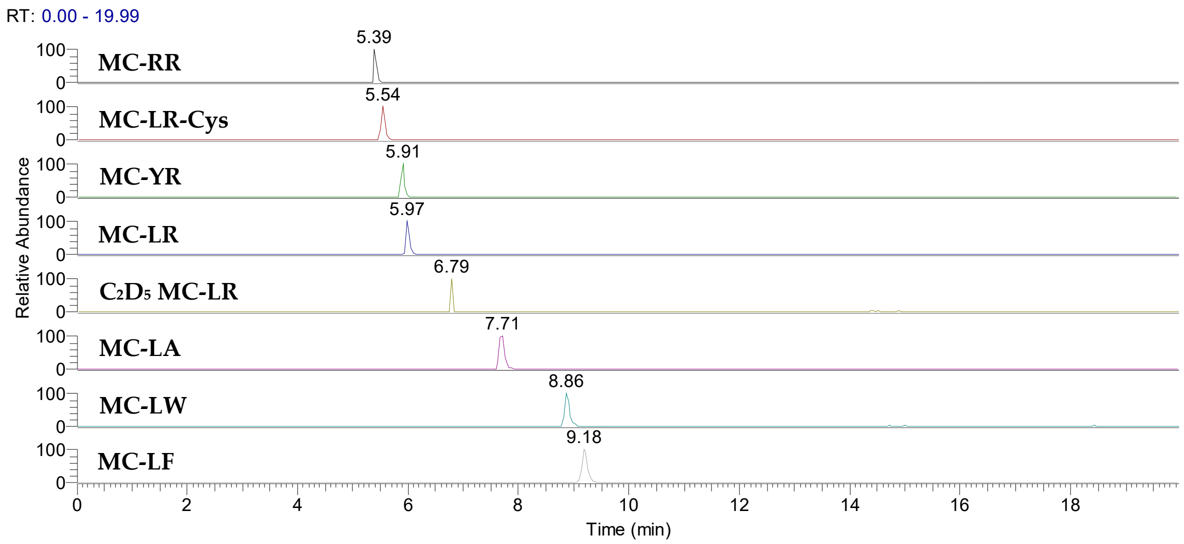

Figure S3. LC-SIM-MS chromatograms showing separation of 8 MCs. *m*/*z* values of detected monoisotopic MC ions are in Table S1.

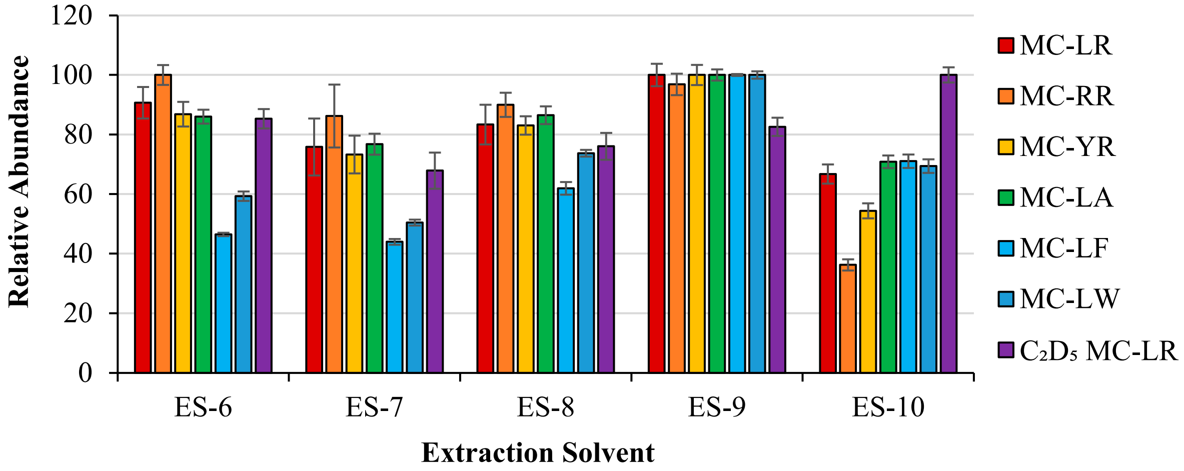

Figure S4. Relative abundances of 7 MCs spiked into 5 mouse liver samples and extracted using 5 different solvents: 55:45 (v:v) $CH_3CN:H_2O$ containing 1% FA (ES-6), 65:35 (v:v) $CH_3CN:H_2O$ containing 1% FA (ES-7), 75:25 (v:v) $CH_3CN:H_2O$ containing 1% FA (ES-8), 85:15 (v:v) $CH_3CN:H_2O$ containing 1% FA (ES-9), and CH_3CN containing 1% FA (ES-10). Relative abundances were compared. Error bars are ± standard deviation of triplicate LC-MS measurements.

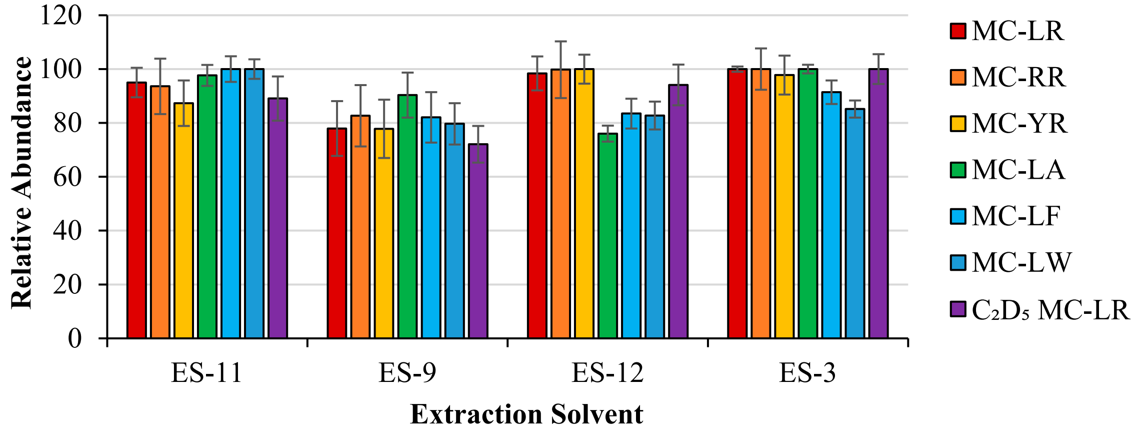

Figure S5. Relative abundances of 7 MCs spiked into 4 mouse liver samples and extracted using 4 different solvents: 85:15 (v:v) CH_3CN:H_2O containing 0.1% FA (ES-11), 85:15 (v:v) CH_3CN:H_2O containing 1% FA (ES-9), 85:15 (v:v) CH_3CN:H_2O containing 100 mM $ZnSO_4$ and 0.1% FA (ES-12), and 85:15 (v:v) CH_3CN:H_2O containing 100 mM $ZnSO_4$ and 1% FA (ES-3). Relative abundances were compared. Error bars are ± standard deviation of triplicate LC-MS measurements.

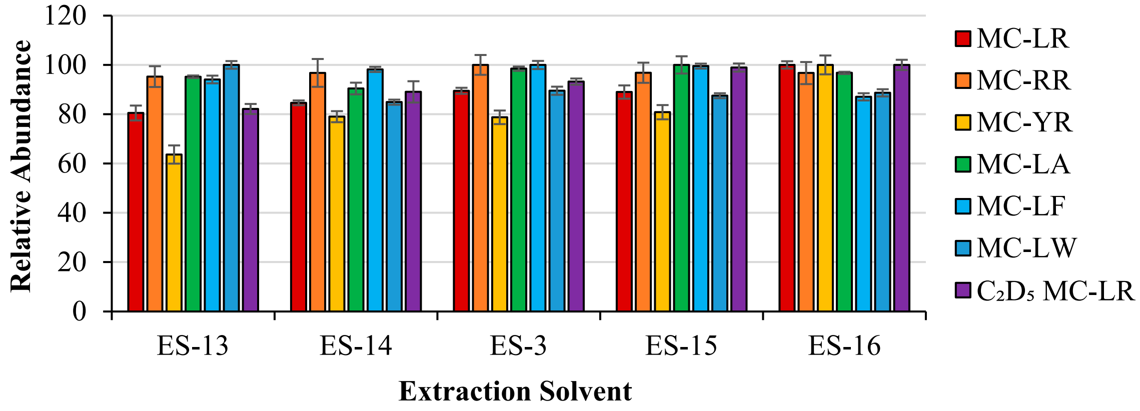

Figure S6. Relative abundances of 7 MCs spiked into 5 mouse liver samples and extracted using 5 different solvents: 85:15 (v:v) CH_3CN:H_2O containing 25 mM $ZnSO_4$ and 1% FA (ES-13), 85:15 (v:v) CH_3CN:H_2O containing 50 mM $ZnSO_4$ and 1% FA (ES-14), 85:15 (v:v) CH_3CN:H_2O containing 100 mM $ZnSO_4$ and 1% FA (ES-3), 85:15 (v:v) CH_3CN:H_2O containing 150 mM $ZnSO_4$ and 1% FA (ES-15), and 85:15 (v:v) CH_3CN:H_2O containing 200 mM $ZnSO_4$ and 1% FA (ES-16). Relative abundances were compared. Error bars are ± standard deviation of triplicate LC-MS measurements.

A)

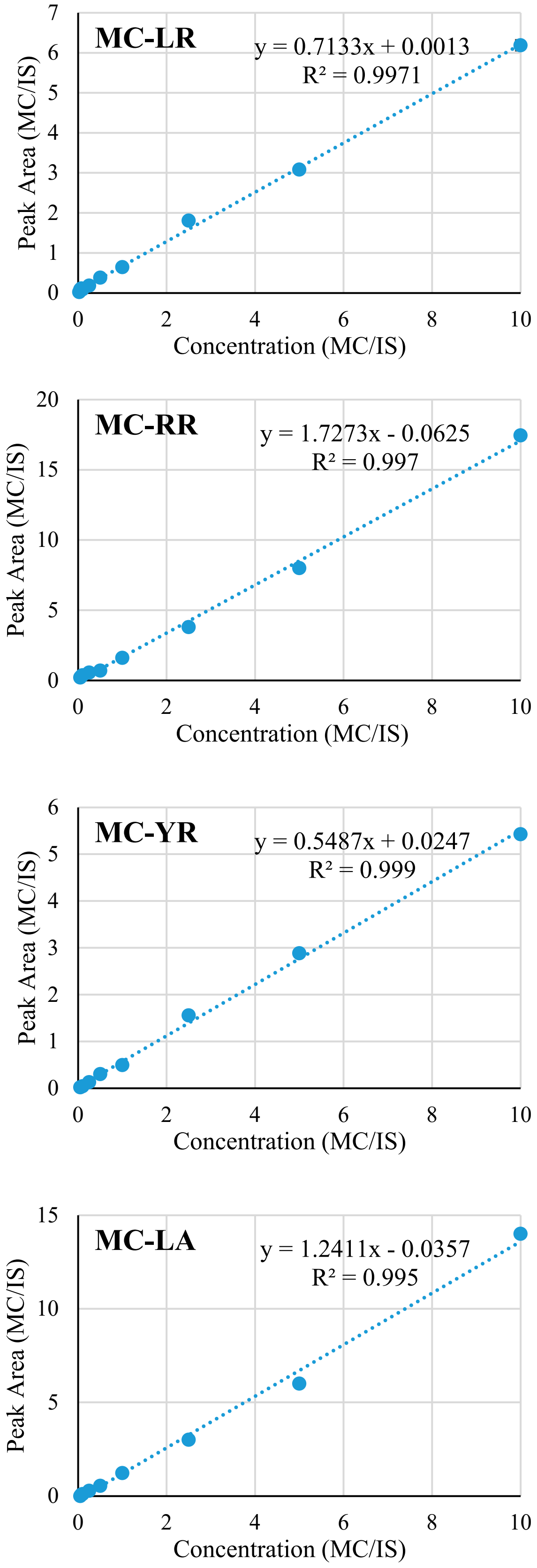
MC-LR
y = 0.7133x + 0.0013
R² = 0.9971
Peak Area (MC/IS)
Concentration (MC/IS)
MC-RR
y = 1.7273x - 0.0625
R² = 0.997
Peak Area (MC/IS)
Concentration (MC/IS)
MC-YR
y = 0.5487x + 0.0247
R² = 0.999
Peak Area (MC/IS)
Concentration (MC/IS)
MC-LA
y = 1.2411x - 0.0357
R² = 0.995
Peak Area (MC/IS)
Concentration (MC/IS)

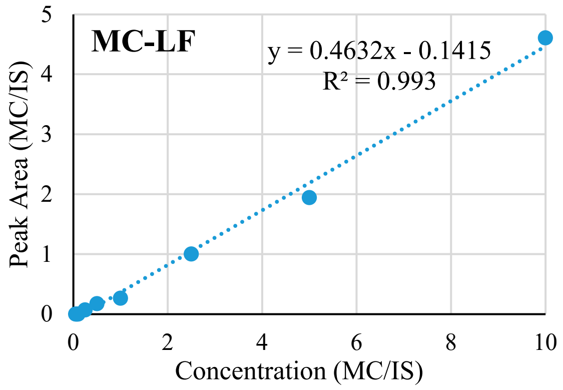
MC-LF
y = 0.4632x - 0.1415
R² = 0.993
Peak Area (MC/IS)
Concentration (MC/IS)
0
1
2
3
4
5
0
2
4
6
8
10

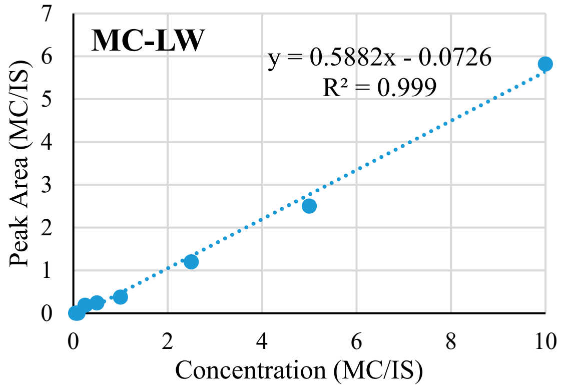
MC-LW
y = 0.5882x - 0.0726
R² = 0.999
Peak Area (MC/IS)
Concentration (MC/IS)
0
1
2
3
4
5
6
7
0
2
4
6
8
10

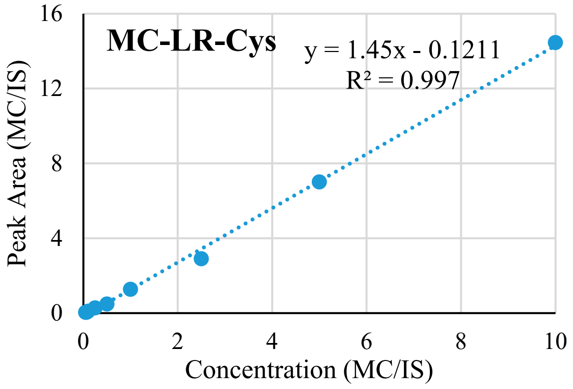
MC-LR-Cys
y = 1.45x - 0.1211
R² = 0.997
Peak Area (MC/IS)
Concentration (MC/IS)
0
4
8
12
16
0
2
4
6
8
10

B)

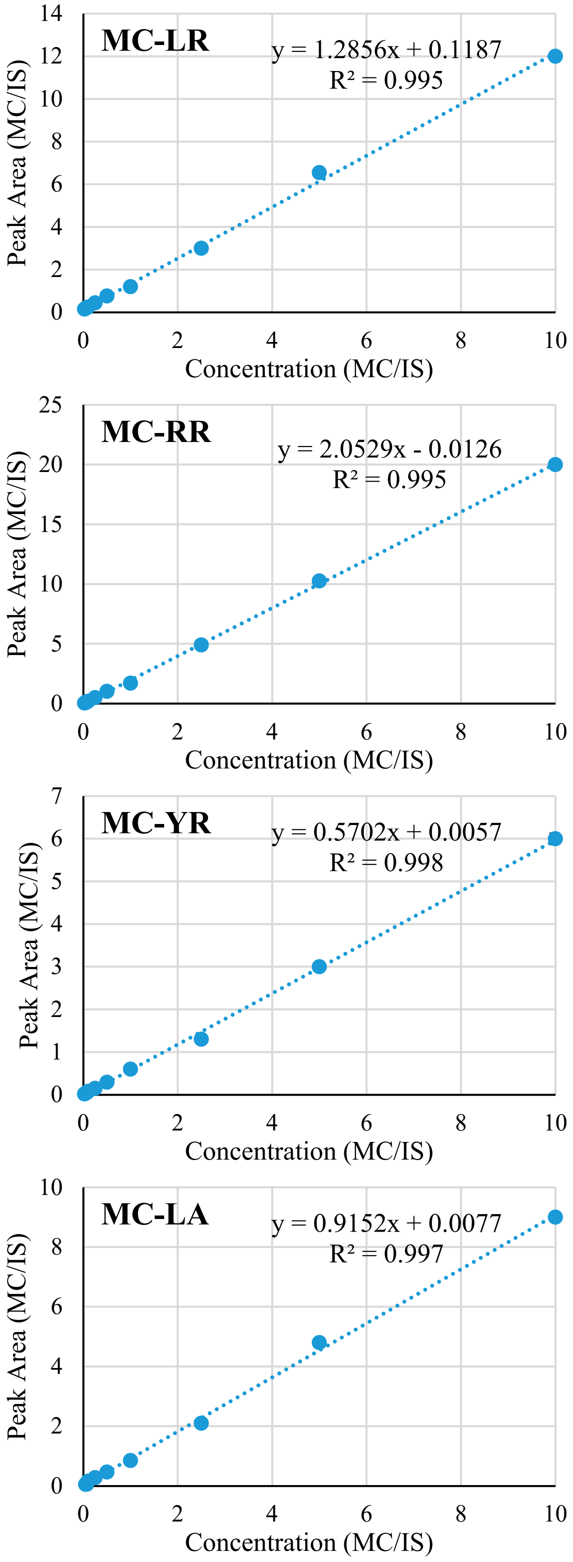
MC-LR
y = 1.2856x + 0.1187
R² = 0.995
Peak Area (MC/IS)
Concentration (MC/IS)
MC-RR
y = 2.0529x - 0.0126
R² = 0.995
Peak Area (MC/IS)
Concentration (MC/IS)
MC-YR
y = 0.5702x + 0.0057
R² = 0.998
Peak Area (MC/IS)
Concentration (MC/IS)
MC-LA
y = 0.9152x + 0.0077
R² = 0.997
Peak Area (MC/IS)
Concentration (MC/IS)

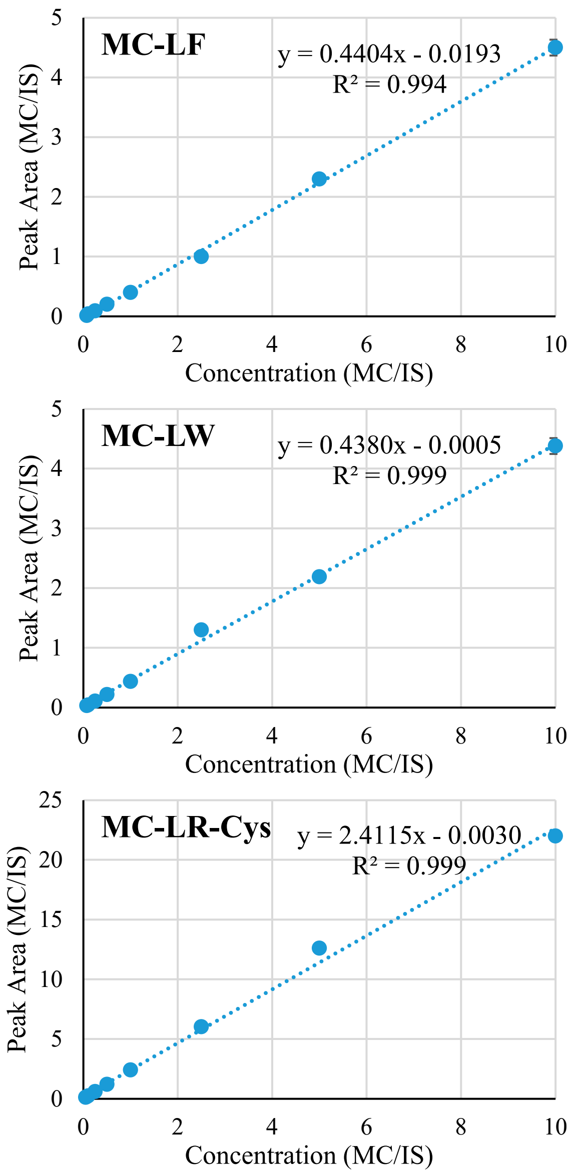

Figure S7. Matrix-matched internal standard (IS) calibration curves used to quantify 7 MCs extracted from **A**) wild-type and **B**) Lepr^{db}/J mouse liver samples.

A)

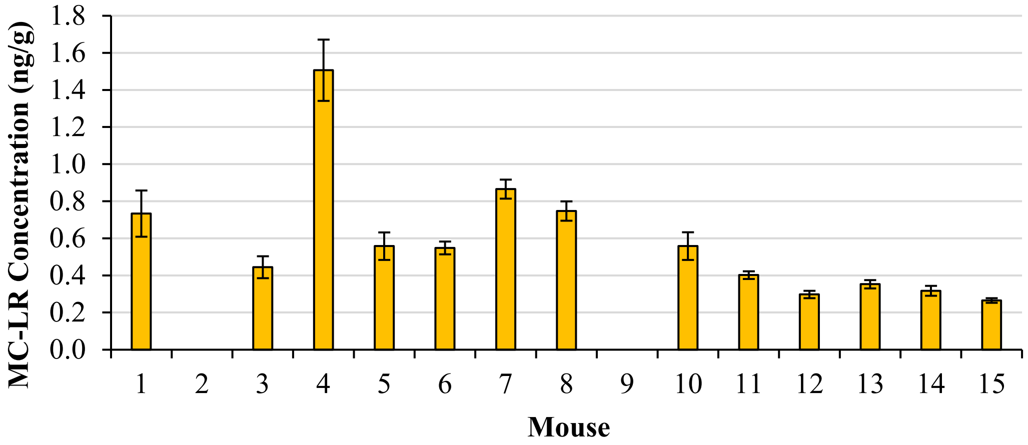

B)

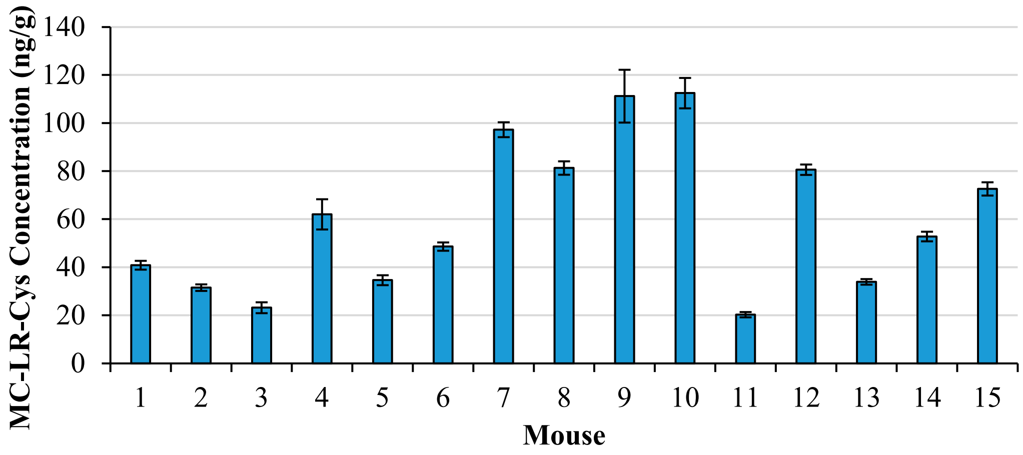

Figure S8. Concentration of **A**) MC-LR and **B**) MC-LR-Cys in the livers of wild-type mice gavaged with 100 µg MC-LR per kg bodyweight. The livers of mice 1–5 were harvested 2 hours after final gavage, the livers of mice 6–10 were harvested 4 hours after final gavage, and the livers of mice 11–15 were harvested 48 hours after final gavage. MCs were extracted from 40-mg liver samples that were spiked with internal standard. MC-LR in the livers of mice 2 and 9 was detected below the LOQ and could not be quantified. Error bars are ± standard deviation of triplicate LC-MS measurements.

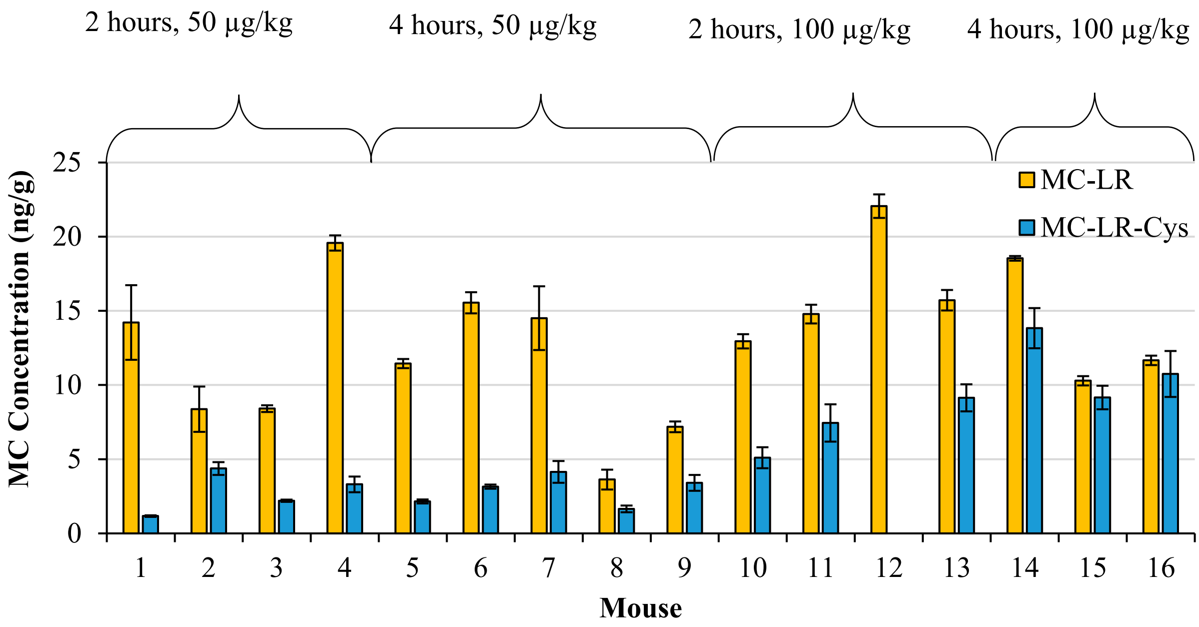

Figure S9. Concentration of MC-LR and MC-LR-Cys in the livers of Lepr^{db}/J mice gavaged with MC-LR. Mice 1–9 were gavaged with 50 µg MC-LR per kg bodyweight, and mice 10–16 were gavaged with 100 µg MC-LR per kg bodyweight. Tissues of mice 1–4 and 10–13 were harvested 2 hours after final gavage, and tissues of mice 5–9 and 14–16 were harvested 4 hours after final gavage. MCs were extracted from 40-mg samples that were spiked with internal standard. MC-LR-Cys concentration in the liver of mouse 12 (45.75 ng/g) was found to be an outlier using Grubbs' test with 95% confidence, and removed. Error bars are ± standard deviation of triplicate LC-MS measurements.

Table S1. Mass accuracies of detected monoisotopic MC ions.

MC Congener	Ion formula	Theoretical *m/z*	Experimental *m/z*	Accuracy (ppm)
MC-LR	$[C_{49}H_{74}N_{10}O_{12} + H]^+$	995.5560	995.5574	1.41
MC-RR	$[C_{49}H_{75}N_{13}O_{12} + 2H]^{2+}$	519.7902	519.7907	0.96
MC-YR	$[C_{52}H_{72}N_{10}O_{13} + H]^+$	1045.5353	1045.5368	1.43
MC-LA	$[C_{46}H_{67}N_7O_{12} + H]^+$	910.4920	910.4935	1.65
MC-LF	$[C_{52}H_{71}N_7O_{12} + H]^+$	986.5233	986.5247	1.42
MC-LW	$[C_{54}H_{72}N_8O_{12} + H]^+$	1025.5342	1025.5356	1.37
MC-LR-Cys	$[C_{52}H_{81}N_{11}O_{14}S + 2H]^{2+}$	558.7915	558.7923	1.43
C_2D_5 MC-LR	$[C_{51}H_{73}D_5N_{10}O_{12} + H]^+$	1028.6187	1028.6200	1.26

Table S2. Percent recoveries of 8 MCs spiked at 2 concentration levels into 20-mg mouse liver samples and extracted using the optimized procedure.

MC Congener	5 ng/g		100 ng/g	
	Recovery (%)	RSD (%)	Recovery (%)	RSD (%)
MC-LR	94.2	2.43	95.0	6.33
MC-RR	92.0	5.35	93.7	3.48
MC-YR	93.7	7.35	95.9	2.25
MC-LA	92.0	5.01	80.6	2.11
MC-LF	80.4	5.35	74.0	1.73
MC-LW	74.0	4.65	60.8	3.12
MC-LR-Cys	71.4	3.11	77.3	2.53
C_2D_5 MC-LR	76.4	3.98	67.9	4.73

Table S3. Percent errors and relative standard deviations at the LOQs of 7 MCs. Calibration curve equations were used to calculate experimental value of LOQs.

MC Congener	LOQ (ng/g)	Experimental value (ng/g)	Error (%)	RSD (%)
MC-LR	0.25	0.29	16.24	13.62
MC-RR	0.50	0.43	13.78	19.81
MC-YR	0.50	0.46	8.72	14.08
MC-LA	0.75	0.82	9.74	3.30
MC-LF	2.50	2.88	15.08	9.11
MC-LW	2.50	2.21	11.63	4.62
MC-LR-Cys	0.75	0.76	1.88	3.15
